# VEGF Receptor Blockade Markedly Reduces Retinal Microglia/Macrophage Infiltration into Laser-Induced CNV

**DOI:** 10.1371/journal.pone.0071808

**Published:** 2013-08-20

**Authors:** Hu Huang, Rachel Parlier, Ji-kui Shen, Gerard A. Lutty, Stanley A. Vinores

**Affiliations:** Wilmer Eye Institute, Johns Hopkins University School of Medicine, Baltimore, Maryland, United States of America; Indiana University College of Medicine, United States of America

## Abstract

Although blocking VEGF has a positive effect in wet age-related macular degeneration (AMD), the effect of blocking its receptors remains unclear. This was an investigation of the effect of VEGF receptor (VEGFR) 1 and/or 2 blockade on retinal microglia/macrophage infiltration in laser-induced choroidal neovascularization (CNV), a model of wet AMD. CNV lesions were isolated by laser capture microdissection at 3, 7, and 14 days after laser and analyzed by RT-PCR and immunofluorescence staining for mRNA and protein expression, respectively. Neutralizing antibodies for VEGFR1 or R2 and the microglia inhibitor minocycline were injected intraperitoneally (IP). Anti-CD11b, CD45 and Iba1 antibodies were used to confirm the cell identity of retinal microglia/macrophage, in the RPE/choroidal flat mounts or retinal cross sections. CD11b(+), CD45(+) or Iba1(+) cells were counted. mRNA of VEGFR1 and its three ligands, PlGF, VEGF-A (VEGF) and VEGF–B, were expressed at all stages, but VEGFR2 were detected only in the late stage. PlGF and VEGF proteins were expressed at 3 and 7 days after laser. Anti-VEGFR1 (MF1) delivered IP 3 days after laser inhibited infiltration of leukocyte populations, largely retinal microglia/macrophage to CNV, while anti-VEGFR2 (DC101) had no effect. At 14 days after laser, both MF1 and DC101 antibodies markedly inhibited retinal microglia/macrophage infiltration into CNV. Therefore, VEGFR1 and R2 play differential roles in the pathogenesis of CNV: VEGFR1 plays a dominant role at 3 days after laser; but both receptors play pivotal roles at 14 days after laser. **In vivo** imaging demonstrated accumulation of GFP-expressing microglia into CNV in both CX3CR1^gfp/gfp^ and CX3CR1^gfp/+^ mice. Minocycline treatment caused a significant increase in lectin^+^ cells in the sub-retinal space anterior to CNV and a decrease in dextran-perfused neovessels compared to controls. Targeting the chemoattractant molecules that regulate trafficking of retinal microglia/macrophage appears to be a compelling therapeutic strategy to control CNV and treat wet AMD.

## Introduction

Choroidal neovascularization (CNV) occurs in exudative or wet age-related macular degeneration (AMD) [1]. The new abnormal blood vessels in CNV sprout from pre-existing choroidal vessels, grow through Bruch’s membrane, and invade the sub-retinal space between the pigmented epithelium (RPE) and the photoreceptor outer segments. Invasion of CNV into the sub-retinal space can cause pathological consequences, including retinal edema, detachment and hemorrhage [2]. CNV development in AMD patients can be characterized into three distinct stages: early/initiation, intermediate/active, and late/involution [3]. The causative factors that trigger CNV formation and the cascades of events during the pathogenesis of CNV are poorly understood, but epidemiological and experimental evidence suggest several risk factors that are associated with CNV formation: genetic pre-disposition, hypertension, cigarette smoking, excessive light exposure, and aging [4]–[7]. To elucidate the mechanisms regulating the pathogenesis of CNV, experimental CNV has been generated in various animal species. The approaches that have been used to create CNV include subretinal deposit of high molecular weight materials, such as matrigel [8] and polyethylene glycol [9], oxidized lipid [10], and laser injury [11], [12]. The first two can be considered comparable to the aberrant deposits of extracellular substance in the sub-retinal space, similar to that present in AMD patients; the third is initiated by damage to Bruch's Membrane and RPE. Despite the differences, both approaches create a microenvironment fostering CNV or angiogenesis in the sub-retinal space and both kinds of models mimic a number of features similar to AMD pathology.

CNV induced by laser injury is essentially a wound healing process that involves at least five pathophysiological components: angiogenesis, inflammation, oxidative stress associated with hypoxia, extracellular matrix deposition, and bone marrow (BM)-derived stem/progenitor cells [13]–[15]. The most damaging of these is angiogenesis (refer to the website: http://www.angio.org/understanding/process.php for details). A number of pro-angiogenic factors, which mediate angiogenesis elsewhere in the eye and body, have also been found to stimulate CNV formation. A partial list of the factors that are involved include VEGF-A and placental growth factor (PlGF) [16]–[18], platelet-derived growth factor (PDGF) -B and –C [19], [20], integrins [21], matrix metalloproteinases (MMP-2 & -9) [22], [23], and toll-like receptor (TLR)-3 [24]. There is an increasing consensus that inflammation is an important mechanism in promoting CNV formation. Three immune responses are known to be involved: bone marrow (BM)-derived leukocytes, the complement system, and retinal microglia. BM-derived leukocytes can be mobilized and home to the CNV site. Macrophages are also implicated in CNV formation in animal models and AMD patients [24]–[26]. The complement system, which is an important component of innate immunity, has been demonstrated to contribute to the pathogenesis of CNV [27], [28]. Accumulating evidence shows that migration of the resident microglia from the inner retina to the sub-retinal space contributes to CNV, as well as other complications of retinal vascular and degenerative diseases [29]–[31]. The precise mechanisms by which these inflammatory cells regulate CNV remain unclear, but two mechanisms were postulated: one is an autocrine mechanism involving direct migration to the lesion and then secretion of pro-inflammatory factors; the other is a paracrine mechanism mediated by stimulating other cells (i.e., RPE) to generate pro-inflammatory factors. Some relevant inflammatory factors include chemokine (C-C motif) ligand 2 (CCL2) [27], complement factor H (CFH) [28], tumor necrosis factor (TNF)-α [14], [32], interleukin (IL)-1β [33]. Also, there is evidence that CNV development is a hypoxic event and is associated with oxidative stress. CNV is almost totally prevented in the absence of the hypoxia response element (HRE) in the VEGF promoter [14]. Oxidative stress markers nitrotyrosine and acrolein are dramatically up-regulated at the CNV lesion, predominantly in RPE [34]. Finally, several studies conducted by Grant and her colleagues show that BM-derived stem/progenitor cells are incorporated into laser-induced CNV and contribute to its formation [15], [35], [36].

Our recent report demonstrated that blockade of VEGFR1 and 2 suppressed pathological angiogenesis in laser-induced CNV, which was associated with an accumulation of GSA-lectin(+)F4/80(+)(CD45(+)CD31(−) cells in the sub-retinal space anterior to CNV [18]. These cells appeared to have impaired migration and failed to infiltrate into CNV due to blockade of VEGFR1 and/or 2. Given that this phenomenon was associated with the reduced CNV formation in the antibody treatment condition, it seems likely that without treatment or in the rat IgG control treatment condition these cells were infiltrating into CNV and interact with the residential cells, such as RPE to make VEGF and other pro-angiogenic factors to stimulate NV. In addition, we suggested the GSA-lectin(+)F4/80(+)CD45(+)CD31(−) cells, which stay in the sub-retinal space anterior to CNV due to VEGFR1 and/or 2 blockade, are likely retinal microglia/macrophage and further hypothesized that their migration and infiltration are regulated by a combination of chemoattractant factors: PlGF-VEGF/VEGFR1 and 2, CCL2/CCR2, SDF1/CXCR4 or CX3CR1. VEGFRs, especially VEGFR1, had been reported to be involved in regulating the recruitment and infiltration of inflammatory cells to pathological sites in different disease conditions. For example, in laser-induced CNV, the deficiency of PlGF, a known ligand for VEGFR1, significantly inhibited infiltration of microglia/macrophage (F4/80^+^ cells) into CNV lesion [37]. In Alzheimer’s disease, VEGFR1 was shown an integral chemotactic component in attracting microglia into neurodegenerative areas in response to amyloid-β peptide [38]. In arthritis and atherosclerosis, anti-FLT1 antibody impaired infiltration of FLT1-expressing leukocytes in inflamed tissues, thereby ameliorating the disease progression [39]. This study was designed to further investigate these observations by examining the distribution of these cells in CNV lesion, particularly on the cross sections. We first showed the differential expression patterns of VEGFR1 and R2 and their three ligands during the pathogenesis of CNV by using the LCM-isolated CNV lesions and surrounding tissues, including RPE, choroid, and neural retina. Next, we showed that at 3 days after laser, blockade of VEGFR1 not R2 influenced CD11b(+), CD45 (+), or Iba1 (+) cells recruitment and that at 14 days after laser, blocking both receptors inhibited the process. Finally, we showed that (i) by using the CX3CR1^gfp/gfp^ mice and fundus photography, in-vivo migration of GFP-labeled retinal microglia into CNV and (ii) direct inhibition of retinal microglia by minocycline significantly suppressed CNV formation, which correlates with the migration inhibition of lectin (+) cells, possibly retinal microglia/macrophage.

## Methods

### Mice

Animal use was in accordance with the approved protocols by the Institutional Animal Care and Use Committee of Johns Hopkins University School of Medicine and the guidelines of the Association for Research in Vision and Ophthalmology. The 6-8-week old C57BL/6 and CX3CR1^gfp/gfp^ mice were purchased from Jackson Lab (Bar Harbor, ME) and housed at the Wilmer Woods Animal Facility of Johns Hopkins University School of Medicine.

### Mouse model of laser-induced CNV

CNV was induced by laser rupture of Bruch’s membrane. C57BL/6J (6-8 week-old) mice were anesthetized with ketamine hydrochloride (100 mg/kg body weight) and xylazine (4 mg/kg body weight) and the pupils were dilated with 1% tropicamide. Laser injury (75 μm spot size, 0.1 sec duration, 120 mW) was performed in the 9, 12, and 3 o’clock positions of the posterior pole of the retina with the slit lamp delivery system of an Oculight GL diode laser (Iridex, Mountain View, CA) and a handheld cover slip as a contact lens to view retina. Only burns in which a bubble was produced were used in the study. 1, 3, 7 and 14 days after rupture of Bruch’s membrane, the mice were anesthetized and the eyes were either embedded in Optimal Cutting Temperature Medium (OCT) compound (Miles Diagnostics, Elkhart, IN) for cryo-sectioning or fixed in 10% buffered formalin for 3 hours for RPE/choroidal flat mounts. At 14 days after laser, mice were perfused by FITC-dextran for the quantification, as previously published procedures [18], [41].

### Laser captures microdissection (LCM), amplified antisense (a) RNA and RT-PCR

LCM was performed with a LMD6000 laser capture microdissection microscope (Leica Microsystems) according to our previously published technique [42]. Briefly, LCM-isolated CNV lesions or surrounding tissues, including RPE, choroid, and neural retina were collected in 60 μl RLT lysis buffer (Qiagen, Valencia, CA) and then stored at −80°C until used. Total RNA was isolated by the RNeasy Micro kit (Qiagen) and then used for cDNA synthesis or aRNA amplification, which was conducted with the SuperScript III first-strand system (Invitrogen, Carlsbad, CA) and Target Amp 2-Round Aminoallyl-aRNA amplification kit (Epicentre, Wilmington, DE), respectively. After digesting the remaining RNA with RNase H, the reaction mix was diluted 5 fold and used as templates in RT-PCR reactions. The housekeeping gene GAPDH was used as PCR controls. The gene-specific primers were the same as those used in our recent publication, which listed the nucleotide sequence of primers [18].

### Antagonists & systemic administration

MF1 and DC101 are rat anti-mouse monoclonal antibodies directed against VEGFR1 and VEGFR2, respectively, that was provided by ImClone Systems (New York, NY), a wholly owned subsidiary of Eli Lilly and Company. The treatment was performed by our recently described schedule: IP injections of 50 mg/kg MF1, 50 mg/kg DC101, or 25 mg/kg MF1+25 mg/kg DC101, and PBS were administered right after laser treatment and followed by every other day (7 treatments in total) [18]. Minocycline was purchased from Sigma-Aldrich (St. Louis, MO), and 50 mg/kg dosages were injected IP immediately after laser treatment and followed by injection every other day (7 treatments by 14 days after laser).

### Immunofluorescence staining, quantification and cell counting

Ten-µm cryosections or choroidal flat mounts were permeabilized with 0.25% Triton X-100 and blocked with 10% goat serum for 1 hr, incubated overnight at 4°C with primary antibodies: rabbit anti-Iba1 for microglia (1∶500, Wako Chemicals, Richmond, VA), mouse anti-CD45 for all leukocytes [1∶100, AbD serotec; Oxford, UK; Developmental Studies Hybridoma Bank (DSHB), Iowa City, IA], mouse anti-CD11b for monocytes (1∶100, DSHB). After rinses in PBS, specimens were incubated with appropriate secondary antibodies at 1∶1000 dilution: Alexa Fluor 488 goat anti-mouse IgG for CD11b and CD45; and Alexa Fluor 594 goat anti-rat IgG for MF1, DC101 and 5D11D4 (an antibody for PlGF); Alexa Fluor 594 goat anti-rabbit IgG for Iba1; and Alexa Fluor 488 donkey anti-goat IgG for VEGF (Invitrogen, Carlsbad, CA). A summary of all the antibodies used is listed in Table 1. For mouse primary antibodies, the anti-mouse secondary antibody was pre-incubated with 0.03 mg/ml normal mouse IgG (Invitrogen, Cat#: 10400C) to prevent from binding to the endogenous mouse IgG, resulting in high background.

**Table 1:** Antibody information.

Antigen	Host	Primary antibody	Dilution	Specimen	Secondary antibody	Source
VEGFR1	Rat	MF1	1/100	Cryo-section	Alexa Fluor 594 goat anti-rat IgG	ImClone System, NYC, NY
VEGFR2	Rat	DC101	1/100	Cryo-section	Alexa Fluor 594 goat anti-rat IgG	ImClone System, NYC, NY
PlGF	Mouse	5D11D4	1/100	Cryo-section	Alexa Fluor 594 goat anti-rat IgG	ThromboGenics, Leuven, Belgium
VEGF-A	Goat	Anti-human VEGF	1/100	Cryo-section	Alexa Fluor 488 donkey anti-goat IgG	R&D Systems, Minneapolis, MN
Ibal1	Rabbit	Anti-Iba1, Rabbit	1/500	Cryo-section & Flat mount	Alexa Fluor 488 goat anti-rabbit IgG	Wako Chemicals, Richmond, VA
CD11b	Mouse	H5A4	1/100	Cryo-section & Flat mount	Alexa Fluor 488 goat anti-mouse IgG	DSHB, Iowa City, IA
CD45	Mouse	H5A5	1/100	Cryo-section & Flat mount	Alexa Fluor 488 goat anti-mouse IgG	DSHB, Iowa City, IA
CD45	Mouse	MCA43GA/OX-1	1/100	Cryo-section & Flat mount	Alexa Fluor 488 goat anti-mouse IgG	AbD serotec, Oxford, UK

For the quantitative comparison of IF images, the specimens and images were prepared to eliminate the errors caused by variations with particular care, as described previously [40]. Briefly, the eye samples of control and experimental groups (4 mice eyes each group) were cryo-preserved in the same module with the superior quarter towards up. The cryo-sections including optical nerves were collected for immunostaining, whose procedures were therefore in parallel with the treatment and control groups. A Zeiss Axioplan2 fluorescent microscopy was used to acquire IF Images with Axion 4 software. The IF-stained specimens were used for quantification within 1 week. The fluorescence intensity and area size of IF images were quantified by ImageJ software. The IF-positive areas were first identified as a region of interest (ROI) by running Image/Adjust/threshold. The mean intensity and area size of ROI were then determined by running Analyze/Analyze particles. The results were averaged from the 4 cryo-sections and then expressed as mean ± SD per section (10 µm).

### Quantifications of digitized immunofluorescent images

Images were acquired with a Zeiss Axioplan2 and quantified by ImageJ (NIH). For the quantitative comparison of drug and control groups, all the procedures were performed in parallel, including preparation of the reagents used for immunostaining, conditions of incubation with antibodies, exposure times while taking pictures, etc. To limit variations, all the measurements were normalized by subtraction of the background fluorescent intensity. For cell counting, the DAPI (+) nuclei with cell body displaying the immune-reactivity of CD11b, CD45 or Iba1 were counted in retina and CNV. The cell number was averaged from the 4 cryo-sections containing CNV and then expressed as a mean ± SD per CNV lesion (10 µm cryosection).

### Fundus Photography and quantification of GFP fluorescent intensity

Microglia migration into CNV was assessed in vivo at various time points post laser with the CX3CR1^gfp/gfp^ mice, in which retinal microglia specifically express the reporter enhanced green fluorescent protein (EGFP). Mice were anesthetized and the pupils were dilated as described above. Fundus photographs of the retina were taken with a Micron III fundus camera (Phoenix Research Lab, Inc., Pleasanton, CA), which allowed us to examine the entire retina including the retinal areas with laser burns. ImageJ was used to measure the mean fluorescence intensity of GFP. Briefly, the GFP-positive microglia cells were selected by adjustment/threshold. The average pixel intensity of laser-burn lesion or normal area was determined by analyzing particles.

### Statistical analyses

Statistical analysis was performed using one-way analysis of variance (ANOVA) or the unpaired Student’s t-test when comparing data from the control to the drug treatment a level of p≤0.05 will be considered statistically significant.

## Results

### Expression of VEGFR1 and R2 and their ligands in the LCM-isolated CNV lesions

**Figure 1:**
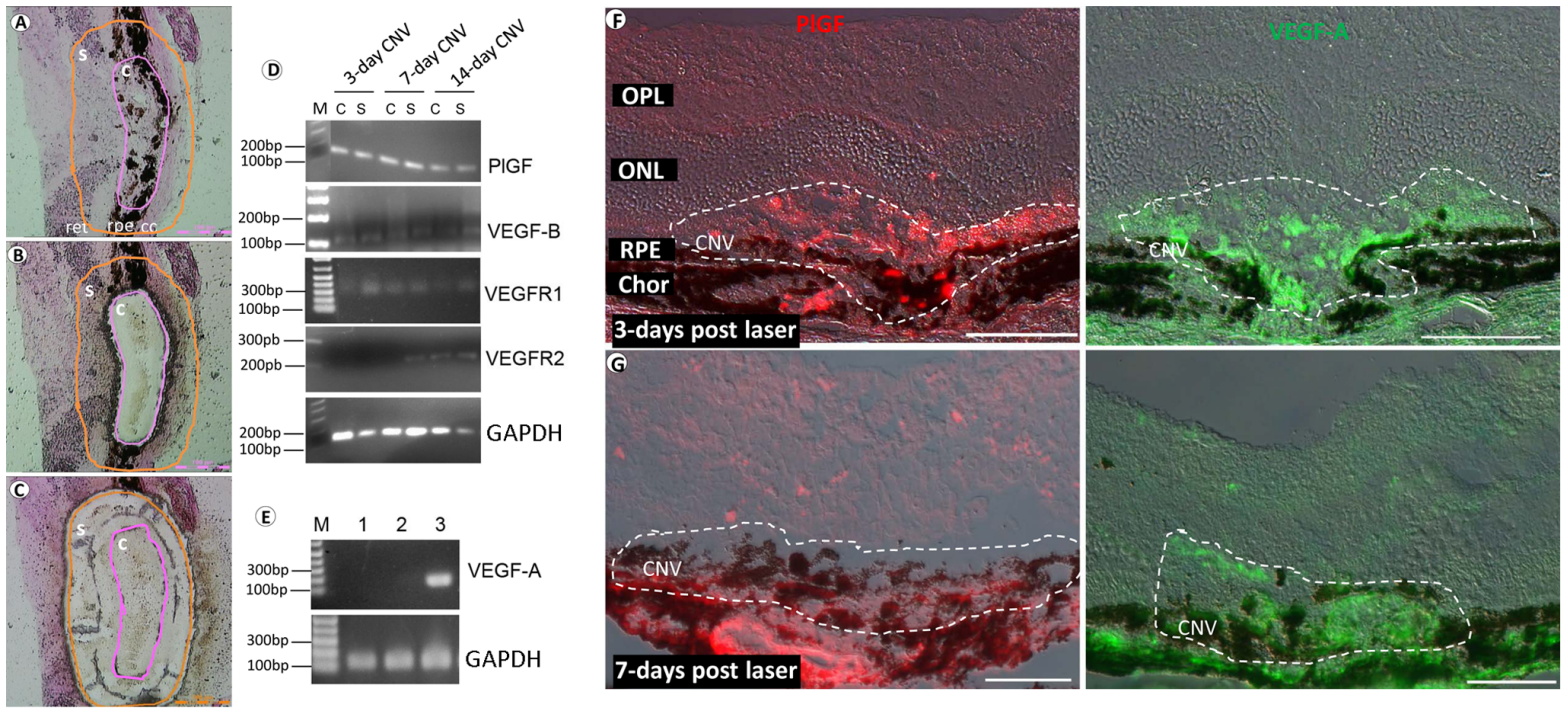
Expression of VEGFR1 and R2 and their ligands in LCM-isolated CNV lesions. (A–C) Representative images showing isolation of the CNV lesions and surrounding tissues by LCM. The central area of CNV lesions (c) are circumscribed by purple lines, while the tissues surrounding CNV lesions (s), including RPE, choroid and neural retina are the areas between the purple and orange lines. (D) PCR results using non-amplified cDNA, which was prepared from the 3, 7, or 14-day LCM-isolated CNV lesions (“c” in A–C) or surrounding tissues (“s” in A–C), as samples. (E) The PCR results of VEGF-A using the amplified aRNAs, which were generated from 3 (lane1), 7 (lane 2), and 14 (lane 3) day CNV lesions, as templates. (F and G) Immunofluorescence (IF) staining of PlGF (left, red) and VEGF-A (right, green) at the 3-day (F, top) and 7-day (G, bottom) CNV. Phase contrast was used to show the retinal layers, RPE and choroid. Chor: choroid. RPE: retinal pigment epithelium. ONL: outer nuclear layer. OPL: outer plexiform layer. Scale bar: 100 µm.

By using the LCM-isolated central area of CNV lesions versus surrounding tissues (Fig. 1A–C), we first examined the spatial and temporal expression patterns of VEGFR1 and 2 and their three ligands PlGF, VEGF-A and VEGF-B at the mRNA level during the pathogenesis of CNV. VEGFR1 and its two ligands PlGF and VEGF-B were detected by RT-PCR in both the central area of CNV lesions and the surrounding tissues in all three stages of CNV development: 3, 7, and 14 day post-laser. VEGFR 2 was not detected in the central area of CNV lesions or the surrounding tissues in 3-day or 7-day CNV lesions, but was positive in the surrounding tissues of 7-day CNV and the CNV lesions and surrounding tissues of 14-day CNV (Fig. 1D). However, we were unable to detect the mRNA transcripts of VEGF-A on the LCM samples, so we generated aRNA from 3-, 7-, 14-day LCM-isolated CNV lesions, as RT-PCR templates. Surprisingly, even with the amplified aRNA samples, VEGF-A was not detected at the 3- and 7-day CNV, but was positive in the 14-day CNV sample (Fig. 1E). We then increased the sample size of 3- and 7-day CNV to 10 and found that there were 6 positives and 4 negatives for VEGF-A mRNA. All the 10 samples of 3- or 7 days CNV were PCR-positive for the housekeeping gene GAPDH, showing the RNAs were not degraded. Despite the variation, which was likely attributed to the extremely-low starting materials from the laser-captured CNV, the results suggested that VEGF-A mRNA is expressed in CNV at the early stages. Furthermore, IF staining of PlGF and VEGF-A showed protein expression at both 3- and 7-day CNV (Fig. 1F & G). These cytokines and receptor signaling molecules, which are likely secreted by activated or proliferative endothelial cells, can trigger the recruitments of inflammatory cells in response to laser treatment. The expression patterns suggested that VEGFR1 and 2 signaling might play roles at different stages in CNV development: VEGFR1 signaling becomes functional starting at the early stages (i.e., 3 days after laser), but VEGFR2 is not present until the relatively late stages (i.e., 14 days after laser). In addition, some other molecules may also be involved in the recruitment process. One example was intercellular adhesion molecule (ICAM)-1. IF staining demonstrated that expression of ICAM-1 was enhanced in the vicinity of CNV (see Figure S1).

### Effects of VEGFR1 and 2 blockade on macrophage activation and recruitment [CD11b (+) cells]

**Figure 2:**
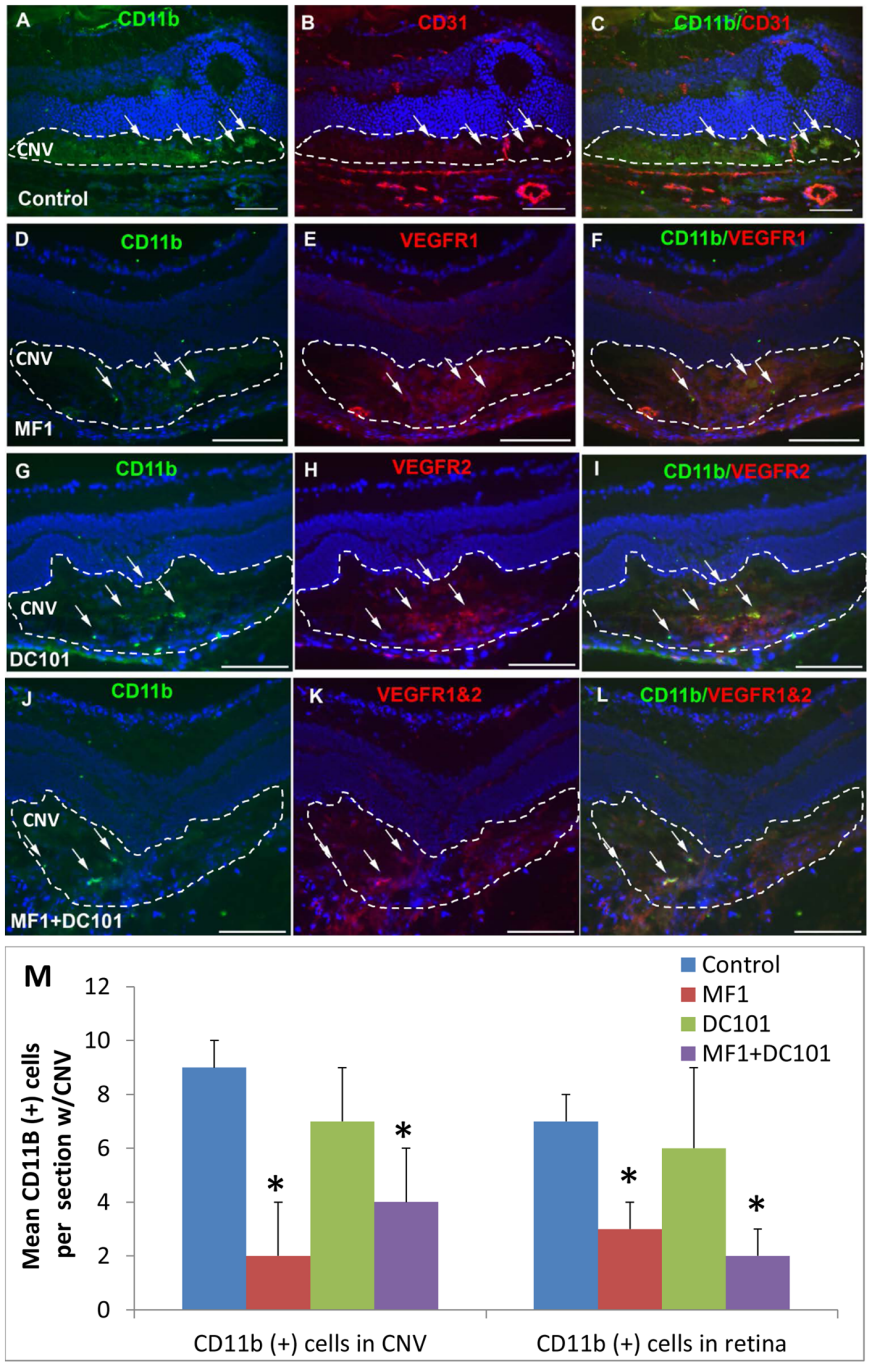
Effects of VEGFR1 and/or 2 blockade on CD11b (+) cells at 3 days after laser. Double labeling of anti-VEGFR1 and/or 2 binding and CD11b was performed on eye cryo-sections of the mice receiving PBS (A–C), MF1 (D–F), DC101 (G–I), or MF1+ DC101 (J–L) treatments. The IF signal of CD11b is shown in panels A, D, G and J; CD31 is in panel B; VEGFR1 and/or 2 are in panels E, H and K; and the merged images are in panels C, F, I and L. (M) The mean CD11b (+) cell number per CNV lesion are expressed as mean ± SD (n = 4).* p<0.05 vs. PBS control. The CNV lesions are circumscribed by hatched lines. Arrows indicate immune-positive cells. Scale bar: 50 µm.

To investigate the roles of VEGFR1 and R2 in regulating retinal microglia/macrophage recruitment to the laser-induced CNV, we asked two questions: 1) whether the cells express the two VEGF receptors; 2) whether the two neutralizing antibodies MF1 and DC101, which were IP injected in our experiments, diffuse into retina and bind to the receptor antigens present on the inflammatory cells, in addition to the new vessels in CNV and the differentiated vasculatures in the retina. In order to address these two questions, CNV was generated in additional mice, which were then divided into one PBS control group (PBS was used as control group because, rat IgG and PBS showed no significant difference in impact on the laser-induced CNV in our previous study) [18] and three antibody treatment groups: MF1 (50 mg/kg), DC101 (50 mg/kg), and MF1+DC101 (25 mg/kg each). RPE/choroid flat mounts were prepared for IF staining with lectin and retinal microglia/macrophage markers at 3 days after laser. In the flat mounts, lectin was present in the CNV lesions from all the groups but, unlike those in 14-day CNV formations where the accumulated lectin (+) cells were segregated (or individual) and countable [18], the lectin (+) cells seen in the flat mounts were numerous and aggregated in the lesion area, making counting difficult. Quantification of fluorescent intensity was also not possible to show significant differences between the PBS control and the three antibody treatment groups (data not shown). Cryosections were prepared for immunofluorescence staining at 3 and 14 days after laser. Double-labeling with antibodies to identify retinal microglia/macrophage and VEGFR1 and/or 2 were performed to show whether MF1 and/or DC101 antibodies were associated with retinal microglia/macrophage. The antibodies to retinal microglia/macrophage markers included anti-CD11b, anti-CD45 (pan leukocyte), and anti-Iba1 (ionized calcium-binding adapter molecule-1 for microglia and macrophage in retina). At 3 days after laser, some CD11b(+) cells were observed in the CNV lesions and nerve fiber layer/ganglion cell layer (NFL/GCL) in the control. In the MF1 treatment group, there was a range from zero to a few CD11b(+) cells in the CNV and the retina. In the DC101 treatment group, the CD11b(+) cells were clearly visible and the cell number was greater than in the MF1 treatment group (Fig. 2G–I); only a few CD11b(+) cells were discernible in the combined treatment of MF1 and DC101, and the cell number was decreased compared to the control or the DC101 treatment (Fig. 2). The mean CD11b(+) cell number per 10 µm CNV area for control was 9±3; for MF1 2±2 (n = 4); for DC101 7±2 (n = 4); for MF1+DC101 was 4±2 (n = 4). The immunoreactivity for VEGFR1, which was due to the perfused MF1 antibody from IP injection (i.e. localization of the injected VEGFR1 antibody), showed that MF1 bound to the VEGFR1+ cells in the CNV and the blood vessels in the retina, whereas VEGFR2 was in CNV but hardly detectable in the retina at this time point (Fig. 2).

**Figure 3:**
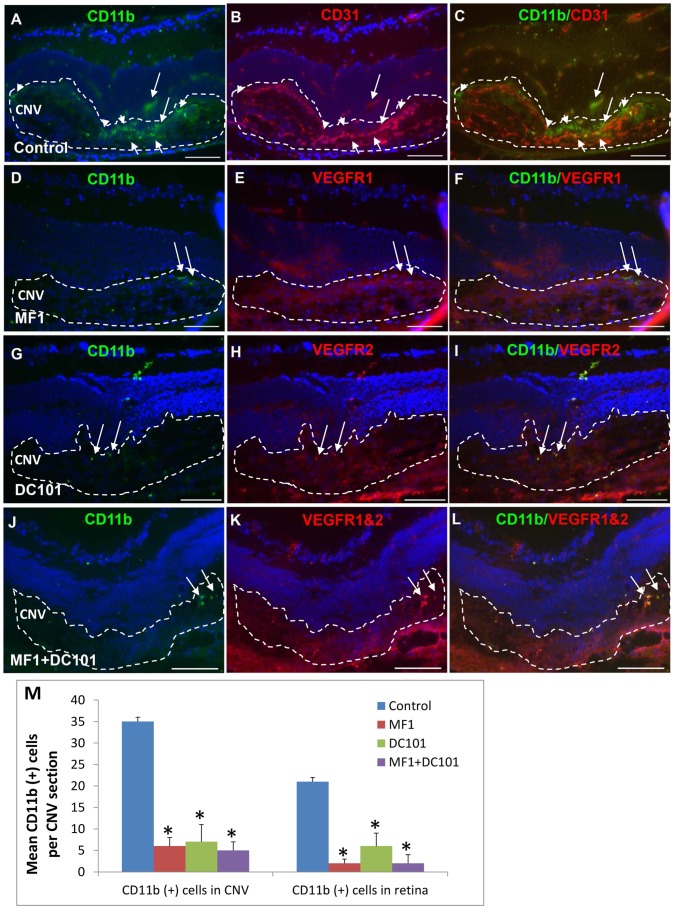
Effects of VEGFR1 and/or 2 blockade on CD11b (+) cells at 14 days after laser. Double labeling of CD11b with CD31 or VEGFR1 and/or 2 was performed on eye cryosections of the mice receiving PBS (A–C), MF1 (D–F), DC101 (G–I) or MF1+ DC101 (J–L). The IF signal of CD11b is shown in panels A, D, G and J; CD31 is in panel B; VEGFR1 and/or 2 are in panels E, H and K; the merged images are in panels C, F, I and L. (M) The mean CD11b (+) cell number per CNV lesion are expressed as mean ± SD (n = 4).* p<0.05 vs. PBS control.The CNV lesions were circumscribed by lines. Arrowheads indicate the CD11b (+) cells are not associated with CD31 (+) blood vessels; arrow indicates the CD11b (+) cells are associated with CD31(+) blood vessels. Scale bar: 50 µm.

At 14 days after laser, a number of CD11b(+) cells were detected in the CNV, as well as scattered throughout the retina from the NFL/GCL to ONL in PBS control. Double labeling of CD11b and CD31 showed that some cells were associated with blood vessels and the others were not (Fig. 3A–C). In the three antibody treatment groups, none or very few of CD11b(+) cells were seen in the CNV or retina. The mean CD11b(+) cell density in the CNV and retina was markedly reduced in the three antibody treatment groups compared to control (Fig. 3D, G, J). The mean CD11b(+) cell number for PBS was 35±6 in the CNV and 21±5 (n = 4) in the retina; for MF1 6±2 (n = 4) in the CNV and 2±1(n = 4) in the retina; for DC101 7±4 (n = 4) in the CNV and 6±3 (n = 4) in the retina; for MF1+DC101 was 5±2 (n = 4) in the CNV and 2±2 (n = 4) in the retina (Fig. 3M). Both mono- and combined treatments of MF1 and DC101 efficiently inhibited CD11b(+) cell recruitment to the outer retina. The immunoreactivity for VEGFR1 or 2 showed that both MF1 and DC101 perfused and bound to the VEGFR1 and 2 antigens on the few CD11b(+) cells present and on blood vessels in the CNV and retina at this late time point (Fig. 3E, H, K).

### Effects of VEGFR1 and 2 blockade on CD45(+) cell recruitment

**Figure 4:**
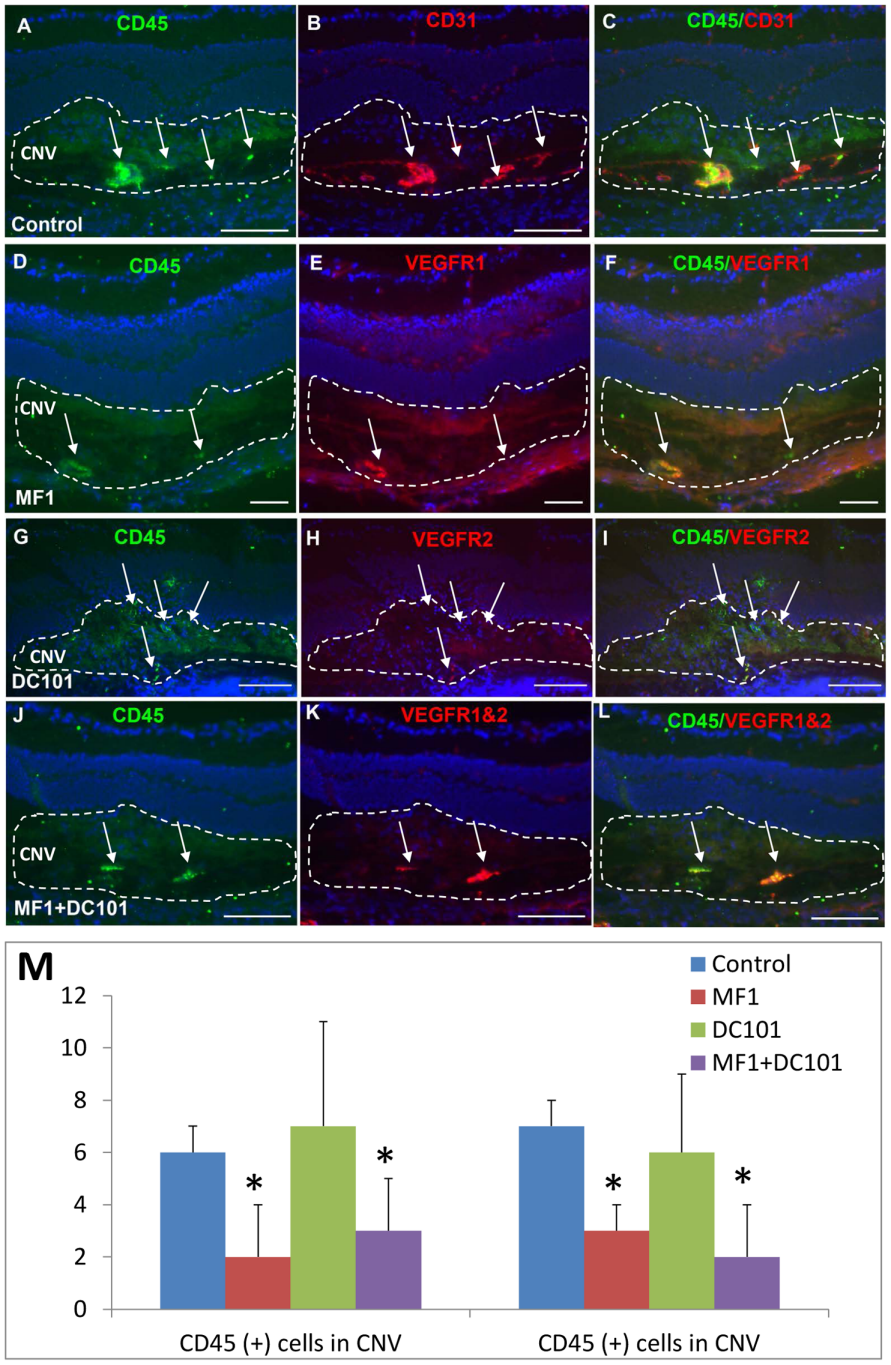
Effects of VEGFR1 and/or 2 blockade on CD45 (+) cells at 3 days after laser. Double labeling of CD45 with CD31 or VEGFR1 and/or 2 was performed on eye cryosections of the mice receiving PBS (A–C), MF1 (D–F), DC101 (G–I) and MF1+ DC101 (J–L). The IF signal of CD45 is shown in panels A, D, G and J; CD31 is in panel B; VEGFR1 and/or 2 are in panels E, H and K; the merged images are in panels C, F, I and L. (M) The mean CD45 (+) cell number per CNV lesion are expressed as mean ± SD (n = 4). * p<0.05 vs PBS control. The CNV lesions are circumscribed by lines. Arrows indicate immune-positive cells. Scale bar: 50 µm.

Immunostaining patterns for CD45 were similar to those for CD11b at both 3 and 14 days after laser. At 3 days after laser, some CD45(+) cells were present in the CNV (Fig. 4). These cells tended to have intense VEGF receptor immunoreactivity and were associated with the CD31(+) blood vessels in the control; range of none to very few CD45(+) cells could be seen in the CNV and retina in the MF1 treatment (Fig. 4D–F). There were more visible CD45(+) cells in the DC101 treatment than in the MF1 treatment and there was an apparent reduction in number in the combined treatment of MF1 and DC101, compared to controls (Fig. 4). The mean CD45 (+) cell number per 10 µm CNV area for control was 6±2; for MF1 2±1 (n = 4); for DC101 7±3 (n = 4); for MF1+DC101 was 3±1 (n = 4).

**Figure 5:**
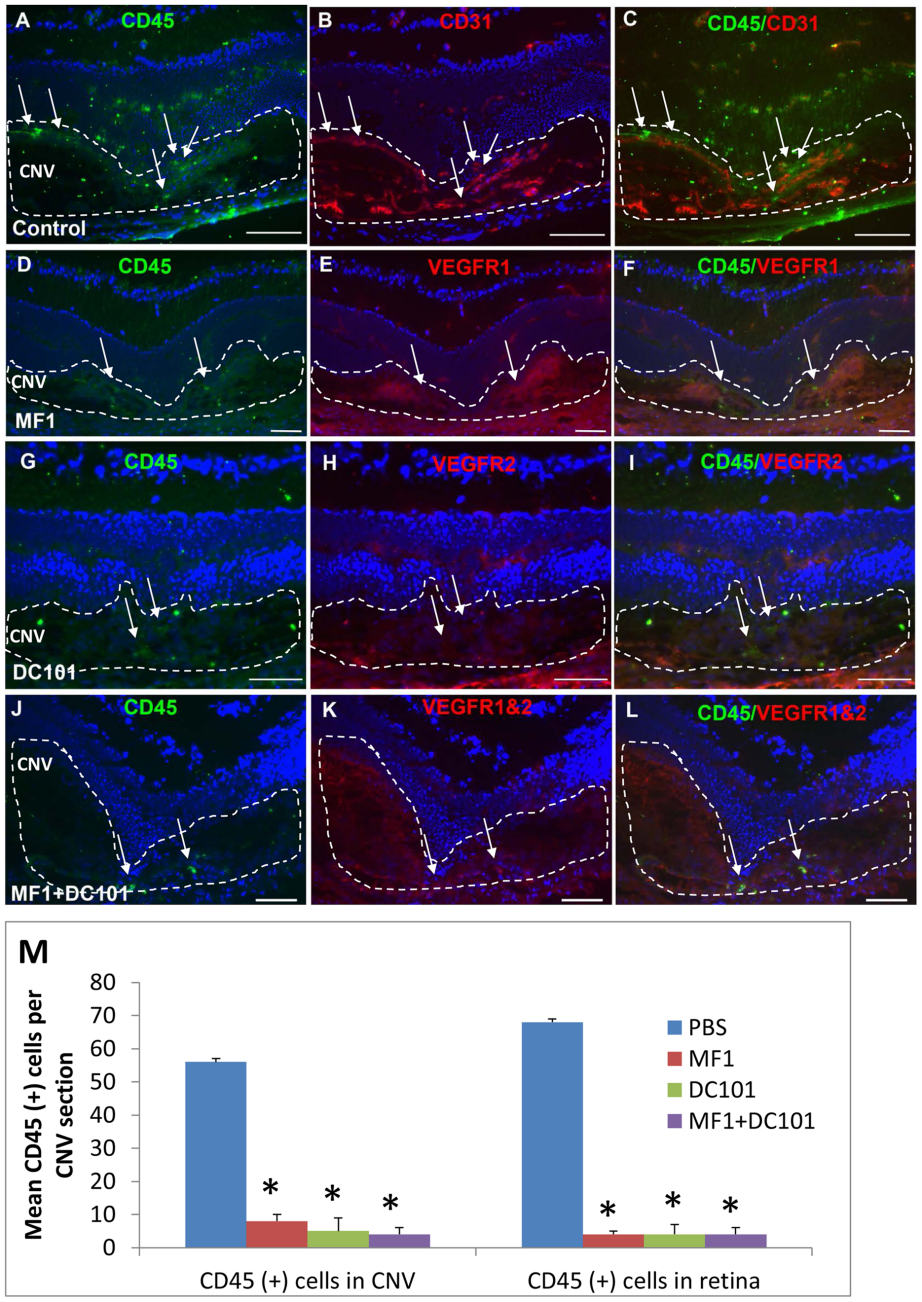
Effects of VEGFR1 and/or 2 blockade on CD45 (+) cells at 14 days after laser. Double labeling of CD45 with CD31 or VEGFR1 and/or 2 was performed on eye cryosections of the mice receiving PBS (A–C), MF1 (D–F), DC101 (G–I) and MF1+ DC101 (J–L). The IF signal of CD45 is shown in panels A, D, G and J; CD31 is in panel B; VEGFR1 and/or 2 are in panels E, H and K; the merged images are in panels C, F, I and L. (M) The mean CD45 (+) cell number per CNV lesion are expressed as mean ± SD (n = 4). * p<0.05 vs PBS control.The CNV lesions are circumscribed by lines. Arrows indicate immune-positive cells. Scale bar: 50 µm.

At 14 days after laser, a number of CD45(+) cells were present in the vehicle-treated specimens; some were recruited to the CNV lesions and the others were scattered throughout the retina from the NFL/GCL to ONL. Double labeling of CD45 and CD31 showed that some cells were associated with blood vessels and some were not (Fig. 5A–C). In the three antibody treatment groups, none to very few of CD45(+) cells were observed in the CNV and the retina. The density of CD45 (+) cells was dramatically decreased in the three antibody treatment groups, compared to PBS control (Fig. 5D, G, J). The mean CD45(+) cell number for control was 56±8 (n = 4) in the CNV and 68±7 (n = 4) in the retina; for MF1 8±3 (n = 4) in the CNV and 4±2 (n = 4) in the retina; for DC101 5±3 (n = 4) in the CNV and 4±3 (n = 4) in the retina; for MF1+DC101 4±2 (n = 4) in the CNV and 2±2 (n = 4) in the retina (Fig. 5M). The mean IF intensity also was significantly reduced in the three antibody treatments, compared to control (data not shown). Both mono and combined treatments of MF1 and DC101 efficiently inhibited the number of CD45(+) cells in the CNV and retina.

### Effect of VEGFR1 and/or 2 blockade on Iba1(+) cell recruitment into CNV

**Figure 6:**
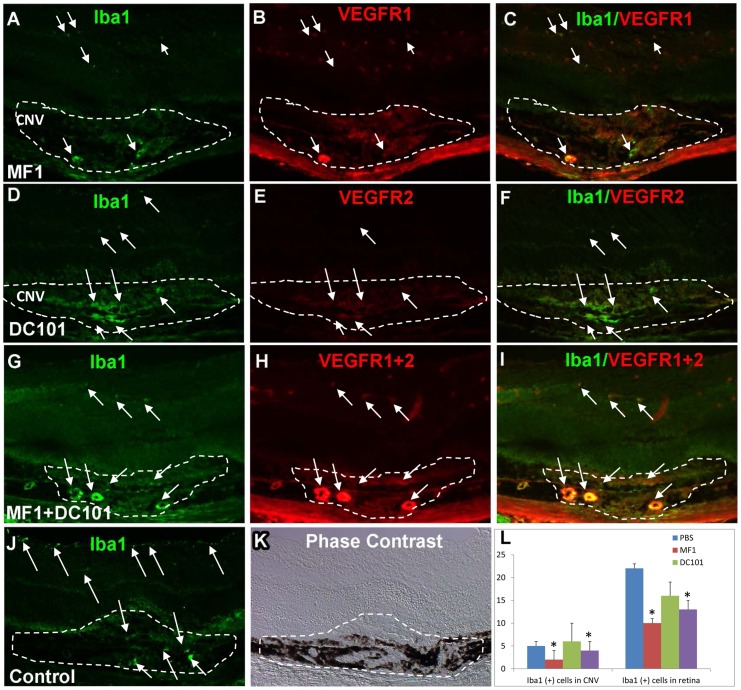
Effects of VEGFR1 and/or 2 blockade on Iba1 (+) cells at 3 days after laser. Immunostaining of Iba1 was performed on retinal cryosections of the mice receiving MF1 (A–C), DC101 (D–E) or PBS (F&G). (H) The mean Iba1 (+) cell number per CNV lesion are expressed as mean ± SD (n = 4). * p<0.05 vs. PBS control. (I) Phase contrast was used defined the CNV lesion. The CNV lesions are circumscribed by lines. Arrows indicate immune-positive cells.

Immunostaining patterns of Iba1 look distinct from those for CD11b or CD45 with regard to the localization and immunoreactivity of VEGF receptors. At 3 days after laser, the Iba1(+) cells were largely present in NFL/GCL and CNV, and some cells were distributed in the inner layer of retina in the PBS control (Fig. 6G). Iba1(+) cells were even sparser and sporadically distributed in the inner retina in MF1-treated specimens and not detected at all in the others (Fig. 6). The mean Iba1(+) cell number was significantly (p = 0.03) less in the MF1 or DC101 treatments than PBS control: 5±2 (n = 4) in the CNV and 22±7 (n = 4) in the retina; for MF1 2±2 (n = 4) in the CNV and 10±3 (n = 4) in the retina; for DC101 6±1 (n = 4) in the CNV and 12±2 (n = 4) in the retina (Fig. 6I).

**Figure 7:**
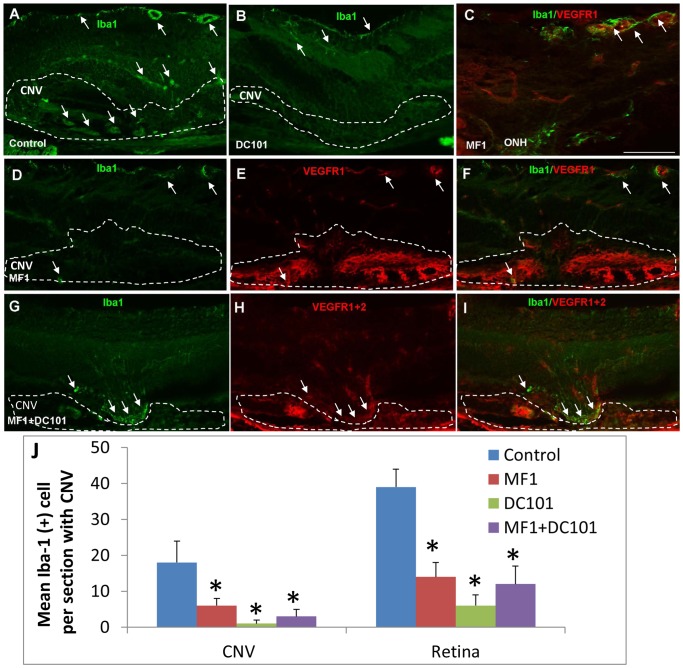
Effects of VEGFR1 and/or 2 blockade on Iba1 (+) cells at 14 days after laser. Single or double labeling of VEGFR1 and/or 2 and Iba1 was performed on eye cryosections of the mice receiving PBS (A), MF1 (B) or MF1+DC101 (C) treatments. (A) In PBS controls, a number of Iba1(+) cells are present in CNV as well as retina. (B) In the DC101 treatment group, Iba1 (+) cells are rare in the CNV; a few of Iba1(+) cells are present in the inner retina. (C) A representative image shows that the Iba1(+) cells localized exclusively to VEGFR1(+) cells and they had a perivascular localization in the inner retina of the mice that were treated with MF1. ONH: optical nerve head. (D–F) In MF1 treatments, very few Iba1 (+) cells are sporadically distributed in the retina and CNV. (G–I) In the MF1 + DC101 treatment group, a cluster of Iba1 (+) cells accumulate in the sub-retinal space anterior to CNV, and are negative or weakly positive for VEGFR1+2. The CNV lesions are circumscribed by dashed lines. Phase contrast was used defined the CNV lesion. (J) Quantification of Iba1 (+) cells in CNV lesions at 14 days after laser. Iba1(+) cells were counted each CNV section (10 μm) from three treatment groups: PBS, MF1 and DC101. The results were expressed as the mean Iba1(+) cell number per CNV lesion ± SD (n = 4). * indicates p<0.05 vs PBS control. Scale bar: 50 µm.

At 14 days after laser, the number of Iba1(+) cells and the immunoreactivity were evidently increased in PBS control, compared to 3 days after laser (Fig. 7). These cells were distributed throughout the retina from NFL/GCL to ONL as well as CNV and had a perivascular localization (Fig. 7A). In the MF1 treatments, the immunoreactivity for Iba1 and the number of Iba1(+) cells were substantially decreased compared to PBS controls; none to very few of Iba1(+) cells were present in the retina or CNV. In the DC101 treatments, a few Iba1(+) cells were in the inner retina (Fig. 7B). The mean Iba1(+) cell number per CNV (10 µm section) for PBS was 18±6 (n = 4) in the CNV and 39±5 (n = 4) in the retina; for MF1 6±2 (n = 4) in the CNV and 2±1 (n = 4) in the retina; for DC101 1±1 (n = 4) in the CNV and 6±3 (n = 4) in the retina (Fig. 7J). The mean IF intensity was also significantly reduced in the MF1 and DC101 treatments, compared to controls (data not shown). In the combined treatment of MF1 + DC101, a cluster of Iba1(+) cells were present in the sub-retinal space, anterior to CNV but did not appear to penetrate within CNV. Double labeling of Iba1 and VEGFR1 and/or 2 demonstrated that Iba1(+) cells were negative or weakly positive for VEGFR1 and/or 2 and rarely showed co-localization with the two receptors (Fig. 7G–I). In addition, as compared with the reports by Santos et al [43] concerning the Iba1(+) cells in the normal differentiated mouse retina, many of the cells in the laser-treated retina were round with very few or no processes and generally localized around the blood vessels. These features suggested laser activated the microglia/macrophage cells.

### Migration of retinal microglia into CNV in vivo and its inhibition by minocycline treatment

**Figure 8:**
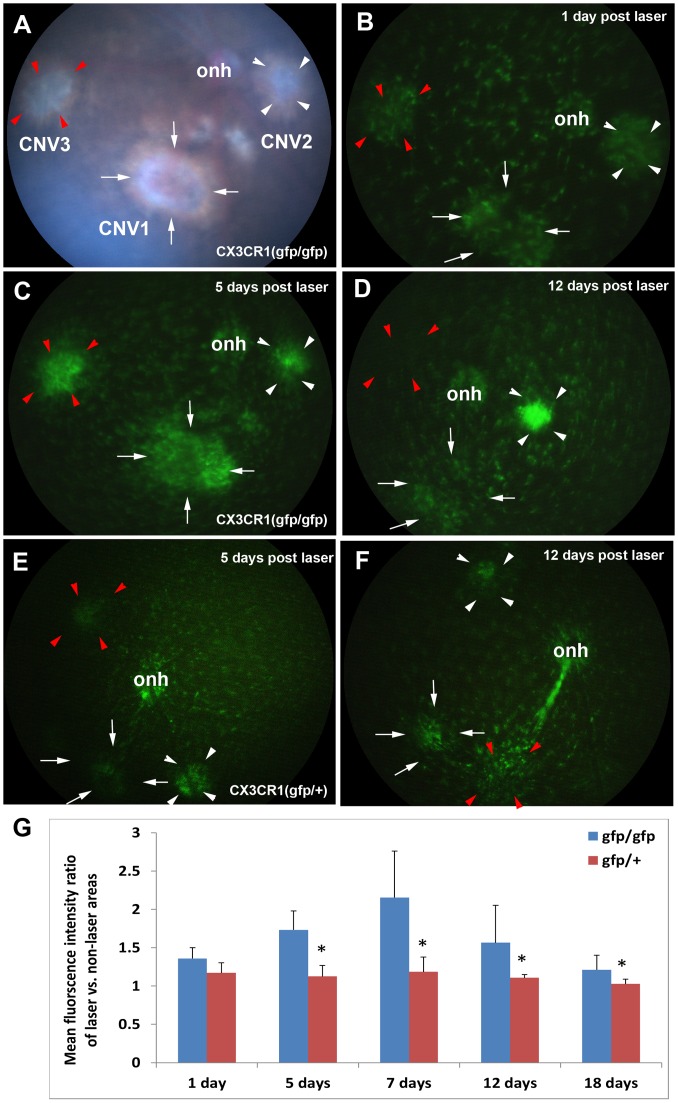
Fundus photography of CX3CR1^gfp/gfp^ and CX3CR1^gfp/+^ mice with CNV. The 3 laser lesions in each eye were indicated by arrows (CNV1), white arrowheads (CNV2) and red arrowheads (CNV3). (A–D) The representative fundus images of CX3CR1^gfp/gfp^ mice with CNV. The same 3 laser burns of one CX3CR1^gfp/gfp^ mouse eye demonstrated the dynamics of GFP-expressing microglia cells during CNV development: 1 days (B), 5 days (C), and 12 days (D) post-laser. (E & F) The representative fundus images of CX3CR1^gfp/+^ mice with CNV at 5 days (E) and 12 days (F) after laser. onh: optical nerve head. (G) The GFP fluorescence intensity ratio of laser vs. non-laser areas showed significant difference between the CX3CR1^gfp/gfp^ and CX3CR1^gfp/+^ at the 5, 7, 12 and 18 days post-laser. * p<0.05.

It has been previously shown that retinal microglia migrate in response to laser in an explant culture or ex vivo model [44]. Here, we used two different approaches to show migration of retinal microglia into CNV in vivo. First, we induced CNV in the CX3CR1^gfp/gfp^ and CX3CR1^gfp/+^ mice, in which microglia specifically express the reporter EGFP, to study microglia migration into CNV in vivo. Fundus photography suggested migration of retinal microglia into CNV: GFP intensity was greater in CNV than non-laser areas. For the homozygous CX3CR1^gfp/gfp^ mice, migration of GFP-labeled microglia was observed at 1 day post laser, had the greatest fluorescent intensity at 5 and 7 days post laser, and then regressed at 12 and 18 days post laser (Fig. 8A–D). Quantification also confirmed the migration of retinal microglia into CNV in response to laser (Fig. 8G). At all the 5 examined time points, the mean pixel intensity of fluorescence was significantly greater in the laser/CNV areas than the non-laser areas (Fig. 8G). At 1 day, the mean was 26.3±3.6 for CNV and 19.4±2.7 for non-laser (p = 0.016 for laser vs. non-laser, n = 10). At 5 days, the mean was 37.2±6.0 for CNV and 27.5±2.4 for non-laser (p<0.0001 for laser vs. non-laser and p = 0.00014 for 5-day vs 1-day laser, n = 10). At 7 days, the mean was 44.7±13.3 for CNV and 20.7±0.4 for non-laser (p<0.034 for laser vs. non-laser; p = 0.053 for 7-day vs 5-day laser, n = 10). At 12 days, the mean was 29.9±10.3 for CNV and 19.9±1.3 for non-laser (p<0.011 for laser vs. non-laser; p = 0.022 for 12-day vs 7-day laser, n = 10). At 18 days, the mean was 23.7±4.6 for CNV and 19.6±1.4 for non-laser (p = 0.02 for laser vs. non-laser; p = 0.23 for 18-day vs 12-day laser, n = 10). Migration of microglia to CNV in response to laser was also observed in the heterozygous CX3CR1^gfp/+^ mice: the mean microglia-GFP intensity is statistically greater in the laser areas than the non-laser areas at the 1-, 5-, 7-, 12-days (but not 18 days) poster laser (Fig. 8E&F). Compared with the CX3CR1^gfp/gfp^ mice, the overall GFP intensity in the retinal fundus of the CX3CR1^gfp/+^ mice was reduced. Unlike the homozygous mice in which the peaking accumulation takes place at the 5 and 7 days after laser, the accumulation of GFP-microglia near CNV in the heterozygous mice was more moderate and consistent without the statistically significant differences during the CNV development. The GFP fluorescence ratio of laser and non-laser areas showed significant differences between the homozygous mice and the heterozygous mice at 5, 7, 12 and 18 days poster laser (Fig. 8G).

**Figure 9:**
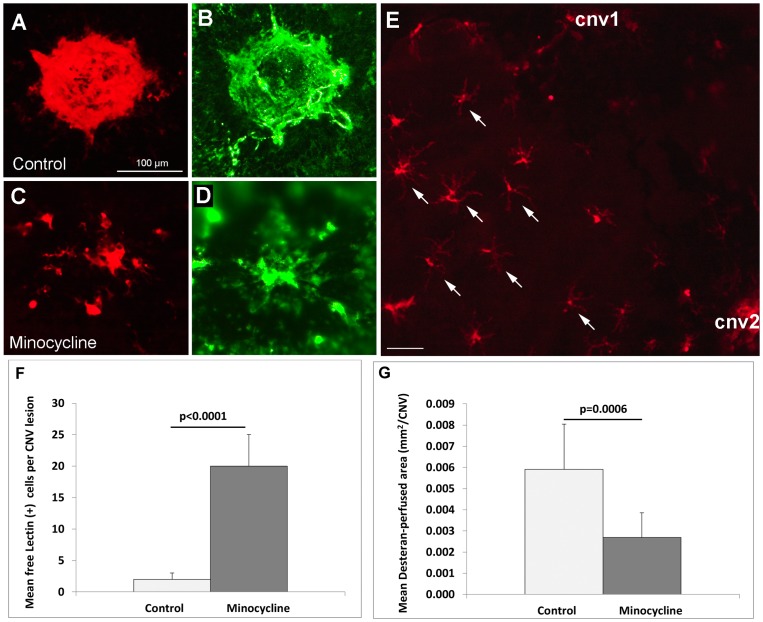
Minocycline treatment led to accumulation of lectin^+^ cells in the sub-retinal space and significantly suppressed laser-induced CNV. (A–D) The representative CNV lesions showed that free lectin (+) cells accumulate in the sub-retinal space anterior to CNV after minocycline treatment (C), but not in controls (A) and that the dextran-perfused neo-vessels are suppressed in the minocycline treatment group (B&D). (E) Microglial/macrophage or lectin (+) cells appeared to have migrated towards the two CNV lesions (CNV1 and CNV2) but were unable to reach the CNV sites after minocycline treatment. Arrows pointed to ramified microglia. (F&G) Quantification shows minocycline led to a significant increase in free lectin(+) cells in the sub-retinal space anterior to CNV (F) and significantly suppressed CNV formation compared to controls (G). Scale bar: 100 µm.

Our second evaluation of the cells as microglia was to treat mice with the compound minocycline, which is known to be a broad spectrum antibiotic as well as an effective inhibitor of microglia/macrophage activation and migration [45]. A marked difference in lectin (+) cells and neovessels was observed between the minocycline treatment and controls: all the lectin-stained CNV (n = 14) from 5 minocycline-treated mice showed individual lectin (+) cells; 4 didn’t show any lectin-positive neovessels at the site of laser injury, 4 showed little lectin-positive neovessels, and 4 showed moderate lectin-positive neovessels (Fig. 9A–D). Only 2 showed strong lectin-positive neovessels of comparable size to WT; whereas all 14 CNV from the 5 control mice eyes showed relatively intense lectin-positive neovessels without or with very few free individual lectin (+) cells (Fig. 9A–D). These observations with minocycline are similar to treatments with MF1(anti-VEGFR1) and/or DC101 (anti-VEGFR2) antibodies in our recent report [18]. In some specimens, lectin (+) cells could be clearly seen to be outside of CNV after minocycline treatment (Fig. 9E). These cells had ramified morphology, suggesting that they were in a non-activated state. The quantitative results (Fig. 9F) showed that minocycline treatment (3x/week, 50 mg/kg) caused a significant increase in individual lectin (+) cells near CNV compared to controls [mean of 20±5/CNV for minocycline and 2±1/CNV for controls (p<0.0001)] and a decrease in dextran-perfused neovessels [mean of 0.0056±0.002 mm^2^/CNV lesion for controls and 0.0027±0.001 mm^2^/CNV lesion for minocycline (p = 0.0006)] at 14 days after laser (Fig. 9G).

## Discussion

Recruitment and accumulation of leukocyte populations like retinal microglia/macrophage in the sub-retinal space contribute significantly to the pathogenesis of AMD [29], [46]. Understanding the mechanisms regulating this recruitment could be significant for the design of AMD therapeutic strategies by targeting microglia/macrophage chemotaxis. In this study, VEGFR1 and 2 were shown to play differential roles in regulating retinal microglia/macrophage infiltration into CNV, depending on the stage of CNV development. VEGFR1 played a dominant role in the early stage, whereas both VEGF receptors play pivotal roles at the late stage with the additive effect of antibody combination. Double labeling suggested that the activated CD11b(+) and recruited CD45(+) cells expressed VEGFR1 but not VEGFR2 at 3 days after laser. However, the cells expressed both receptors at 14 days after laser. Iba1(+) cells tended to be negative or weakly positive for VEGFR1 and/or 2 (see Fig. 7I). The three leukocyte antibodies suggested that some of the inflammatory cells were retinal microglia/macrophage, since Iba1 labels retinal microglia/macrophage, CD11b labels the monocytes and monocyte-derived cells (upregulated upon activation), and CD45 labels all leukocytes. Fundus photography of CX3CR1^gfp/gfp^ and CX3CR1^gfp/+^ mice clearly demonstrated the migration of retinal microglia into CNV lesions in vivo. A previous report showed retina microglia migrated towards laser injury in an explant culture model [44]. Furthermore, the FDA-approved drug minocycline effectively inhibited CNV formation and prevented the accumulation of retinal microglia/macrophage. Despite these findings, we could not rule out the possibility that the reduced infiltration of retinal microglia/macrophage into CNV by VEGF receptor blockade was the indirect effect following inhibition of pathological angiogenesis. These results suggested that it is mostly likely that both two mechanisms that are involved in this process. First, at the earlier stages when neovascularization (NV) has not formed yet, such as 3 days post-laser, the inhibitory effects of VEGFRs blockade on recruitment of inflammatory cells to sites of CNV is an independent event. Since VEGFR2 isn’t expressed in CNV at the earlier stages, the inhibitory effects are mostly likely attributed to VEGFR1 and not VEGFR2 blockade. The later stage when NV starts forming, such as 7 and 14 days post-laser, the inhibitory effects are likely attributed to not only the direct inhibition and but also the reduced neovascularization. Because both VEGFR1 and VEGFR2 are expressed at the later stages, the angiogeneic response involves both receptors. So inhibition of infiltration directly inhibits angiogenesis, which suggests that the infiltrating cells make VEGF to stimulate angiogenesis. In vitro assays on retinal microglia/macrophage migration may provide further evidence.

In our previous report, we found that inflammatory cells, which were immmuno-positive for lectin, CD45 and F4/80, remained in the sub-retinal space anterior to CNV lesion after VEGFR1 and 2 blockade. We hypothesized that these cells were largely retinal microglia, which were activated by laser and migrated from the inner retina [18]. In the present study, a cluster of Iba1(+) cells were found to migrate and accumulate in the area superficial to the CNV and didn’t penetrate into the CNV after VEGFR1 and 2 blockade or by the combined treatment of MF1 and DC101 (Fig. 7G–I). These results appear to be consistent with our hypothesis, but we do not rule out the possibility that some of these accumulated cells are other types of leukocytes. Furthermore, double labeling of Iba1 with VEGFR1 and/or 2 demonstrated that the Iba1(+) cells were generally negative or very weak for VEGFR1 or 2. In contrast, the CD11b(+) or CD45(+) cells were always positive for VEGFR1 or 2. The two sub-populations: VEGFR1&2(+)CD45(+)CD11b(+) and VEGFR1&2 (−)Iba1(+), could be activated and recruited to CNV. Therefore, one could rationalize that VEGFR1& 2 may directly regulate CD11b(+) CD45(+) cell activation and migration but indirectly affect Iba1(+) subset of cells, which may rely on some other chemoattractant factors, such as CX3CR1. Furthermore, it’s possible that the former represents the blood-derived monocytes since VEGF/VEGFR1 signaling was suggested to be the mediator of activation and migration of human monocytes [47] and that the latter represents the retinal microglia, which have other origin and are independent of VEGF receptor signaling [31]. One potential outcome of preventing these immune inflammatory cells from being recruited to CNV is that retinal microglia would not interact with RPE, perhaps preventing RPE from secreting pro-angiogenic and pro-inflammatory factors, which has been suggested to contribute to CNV formation [30]. Another potential outcome is a disruption of communication between retinal microglia and angiogenic sprouts, leading to suppression of angiogenic growth, a mechanism demonstrated to regulate angiogenesis [31]. It is noteworthy that a transient accumulation of retinal microglia was also observed in the CX3CR1-deficient mice, but unlike the outcome after blocking VEGFR1and 2 signaling, CNV was exacerbated. This suggests that, unlike blockade of VEGFR1 and/or 2, retinal microglia ingress into CNV lesions and promotion of CNV formation is due to CX3CR1 deficiency [29]. Given that CX3CR1 plays an indispensible role in the migration of microglia [29], the migration capability of retinal microglia cells are expected to be different between the homozygous CX3CR1^gfp/gfp^ mice and the heterozygous CX3CR1^gfp/+^mice because the two genetic alleles of Cx3cr1 gene were replaced with gfp in the homozygous mice and only one allele was replaced with gfp in the heterozygous mice. Under fundus microscope, it is evident that the retina of homozygous mouse had much greater GFP intensity than the retina of heterozygous retina. By quantifying the fundus images, we found 1) that GFP intensity is significantly greater in the laser burn areas than the non-laser burn areas in both genotypes and 2) that the fluorescence intensity ratio of laser and non-laser areas was significantly greater in the homozygous than the heterozygous. The first finding suggests that, although the migration ability of GFP-labeled microglia is impaired due to PGF deficiency, these GFP-cells migrate to the laser-burn area in response to laser injury, which is likely driven by other chemoattractant factors rather than CX3CR1. The second finding suggests that CX3CR1 plays a greater role in the exiting (egression) than recruitment (ingression) of the microglia, thereby leading to the overstay in the homozygous compared to the heterozygous.

It is believed that correlation of spatiotemporal expression patterns with physiopathological conditions provides crucial clues for the function of genes or proteins of interest in the diseases. For example, one study showed the time dependence of membrane attack complex (MAC), CCL2 and VEGF expression in CNV development and postulated that the cascade of laser inducing MAC and then CCL2 caused up-regulation of VEGF and then formation of CNV. This relationship or interaction was supported by a “loss-of-function” strategy with respective neutralizing antibodies in laser-induced CNV [48]. Two other studies from Li’s lab showed the up-regulation of PDGF-BB and -CC after laser treatment was crucial in modulation of CNV formation [19], [20]. Despite these advances, studies on genes expressed in CNV are predominantly limited to single genes or a few genes of interest with the extracts of the whole retina or cross-sections from CNV lesions [49], [50]. To our knowledge, only a few published reports utilized large-scale gene expression to study the effects of laser photocoagulation on the retina, but these studies dealt with the profound effects of laser on retinas and the identified genes that are associated with various functions of retina [51], [52]. Moreover, a number of genes and proteins are found to be contributors to CNV pathogenesis, but what the spatial and temporal patterns of their expression are and what cell types produce them in CNV development are still poorly understood. So it would be necessary to investigate the patterns of gene profiles expressed specifically by the cells of CNV, which can be achieved by LCM and high throughput expression assays such as next generation or deep RNA sequencing.

In conclusion, VEGF receptor blockade inhibits white cell presence in laser-induced CNV. Two sub-populations of white blood cells appear to be affected: VEGFR1&2(+)CD45(+)CD11b(+), which represent circulating cells responding to laser injury, and VEGFR1&2 (−)Iba1(+), which represent the microglia in retina. It is possible that the former responds to the receptor antibody therapy and cease in their quiescent state to produce cytokines like CX3CR1 that are needed to recruit the latter. This strategy and inhibition of microglia/macrophage migration with minocycline resulted in less CNV. Therefore, targeting infiltration of retinal microglia/macrophage and/or other leukocytes is a potential therapeutic strategy for treating CNV in AMD.

## Supporting Information

Figure S1 **Double labeling of Immunofluorescence (IF) staining of ICAM-1 and CD31.** (A) ICAM-1. (B) CD31. (C) The merged image showed the co-localization of ICAM-1 and CD31. Arrows pointed to the both positive cells for ICAM-1 and CD31. The cryo-section was prepared from the eye with CNV at 14 days post laser.
(TIF)
