# Supplementary figures and images for: VEGF Receptor Blockade Markedly Reduces Retinal Microglia/Macrophage Infiltration into Laser-Induced CNV
Source: PLoS One. 2013 Aug 20;8(8):e71808. doi: 10.1371/journal.pone.0071808 (PMC3748119; doi:10.1371/journal.pone.0071808)

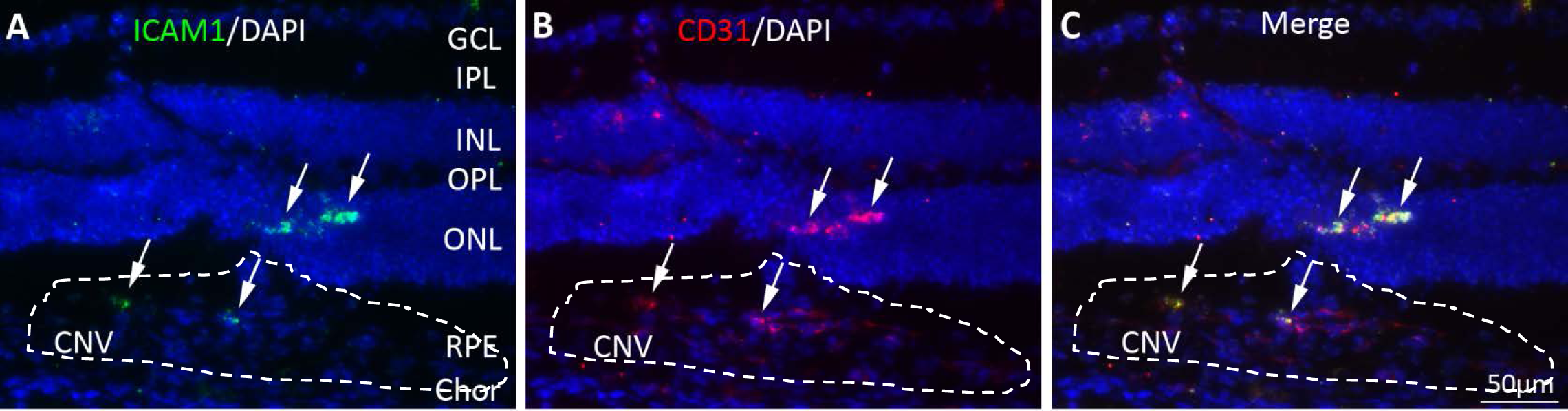

Supplement: Figure S1 — Double labeling of Immunofluorescence (IF) staining of ICAM-1 and CD31. (A) ICAM-1. (B) CD31. (C) The merged image showed the co-localization of ICAM-1 and CD31. Arrows pointed to the both positive cells for ICAM-1 and CD31. The cryo-section was prepared from the eye with CNV at 14 days post laser. (TIF) [file pone.0071808.s001.tif]
